# A Lipid Perspective on Regulated Pyroptosis

**DOI:** 10.7150/ijbs.81017

**Published:** 2023-04-25

**Authors:** Yun Qiu, Ya-Ning Shi, Neng Zhu, Shuo Zhang, Chan-Juan Zhang, Jia Gu, Peng He, Ai-Guo Dai, Li Qin

**Affiliations:** 1 Laboratory of Stem Cell Regulation with Chinese Medicine and Its Application, School of Pharmacy, Hunan University of Chinese Medicine, Changsha 410208, China.; 2 Science and Technology Innovation Center, Hunan University of Chinese Medicine, 410208, Changsha, Hunan, China.; 3 Department of Urology, The First Hospital of Hunan University of Chinese Medicine, Changsha 410208, Hunan, China.; 4 Hunan Provincial Key Laboratory of Vascular Biology and Translational Medicine, Changsha 410208, Hunan, China.; 5 Department of Respiratory Medicine, First Affiliated Hospital, Hunan University of Chinese Medicine, Changsha 410021, Hunan, China.

**Keywords:** pyroptosis, lipids, lipid metabolism, NLRP3, inflammasome.

## Abstract

Pyroptosis is a novel pro-inflammatory cell programmed death dependent on Gasdermin (GSMD) family-mediated membrane pore formation and subsequent cell lysis, accompanied by the release of inflammatory factors and expanding inflammation in multiple tissues. All of these processes have impacts on a variety of metabolic disorders. Dysregulation of lipid metabolism is one of the most prominent metabolic alterations in many diseases, including the liver, cardiovascular system, and autoimmune diseases. Lipid metabolism produces many bioactive lipid molecules, which are important triggers and endogenous regulators of pyroptosis. Bioactive lipid molecules promote pyroptosis through intrinsic pathways involving reactive oxygen species (ROS) production, endoplasmic reticulum (ER) stress, mitochondrial dysfunction, lysosomal disruption, and the expression of related molecules. Pyroptosis can also be regulated during the processes of lipid metabolism, including lipid uptake and transport, de novo synthesis, lipid storage, and lipid peroxidation. Taken together, understanding the correlation between lipid molecules such as cholesterol and fatty acids and pyroptosis during metabolic processes can help to gain insight into the pathogenesis of many diseases and develop effective strategies from the perspective of pyroptosis.

## Introduction

Pyroptosis is involved in essential innate immunomodulatory mechanism and originally defined as caspase-1-dependent programmed cell death (PCD) by Cookson BT and Brennan MA in 2001 1. Pyroptosis occurs primarily in monocytes, macrophages, and dendritic cells, but can also be detected in other cell types, such as endothelial cells, cardiomyocytes, and hepatocytes 2-4. Pyroptosis is driven by inflammatory caspase‐1, caspase‐4, caspase‐5 and caspase‐11, and executed by the gasdermin (GSDM) family. GSDMs induce membrane rupture and the release of intracellular contents, such as interleukin-1β (IL-1β), IL-18, high mobility group box 1 (HMGB1) and Adenosine triphosphate (ATP), which trigger local or systemic inflammatory responses 5. Studies demonstrate that pyroptosis has a dual effect on organisms. Mild-to-moderate pyroptosis can accelerate the immune response by recruiting more immune cells to clear endogenous or exogenous danger signals (e.g., oxidative stress, hyperglycemia, inflammation), effectively prevent excessive proliferation of cells, and maintain the normal development and homeostasis of organisms 6, 7. However, exaggerated pyroptosis leads to an overwhelming inflammatory response 8, to some extent resulting in tissue damage 7, thereby exacerbating disease progression and outcomes, such as cardiovascular diseases, liver diseases, and nervous system diseases 9-11.

Lipids are one of the major nutrients in humans and are pivotal for maintaining cellular structure as well as providing energy and mediating signaling transduction. Lipid biosynthesis and catabolism produce a variety of biological mediators, which are biologically active lipid molecules (also known as signaling molecules or second messengers) that regulate versatile signaling pathways. Previous studies by our group have identified an association between inflammation and lipid accumulation, as well as lipid-triggered inflammatory reactions 12, 13. In addition, pyroptosis is a form of inflammatory cell death. Recent studies have revealed that lipid metabolism may induce pyroptosis during the process of diseases, suggesting a close relationship between lipid metabolism and pyroptosis. However, the molecular mechanism between lipid and pyroptosis remains largely unclear.

### The Mechanism and Features of Pyroptosis

Generally, gasdermin family members are core among the pyroptosis pathway, which can be cleaved and activated by inflammatory caspase. There are six members of gasdermin family in human, including GSDMA, GSDMB, GSDMC, GSDMD, GSDME and DFNB59. Except for DFNB59, other gasdermins consist of a pore-forming N-terminal (NT) domain and a C-terminal (CT) regulatory domain, and the NT of gasdermins induce pyroptosis. Among these proteins, GSDMD is extensively studied in pyroptosis and has been identified as the executioner of pyroptosis 14-16.

Pyroptosis can be divided into the canonical inflammasome pathway and the noncanonical inflammasome pathway. Canonical pyroptotic death is mediated by inflammasome assembly 5. When the host is resistant to microbial infection, multimolecular complexes called inflammasomes will be activated. In addition, inflammasomes are also associated with non-microbial diseases. Accumulating evidence shows that intracellular risks and environmental stimuli, such as cholesterol crystals (CHCs), oxidized LDL (ox-LDL), uric acid crystals, saturated fatty acids (SFA), DNA, mitochondrial ROS and lysophosphatidylcholine (LPC), can lead to the assembly of inflammasome 17. Most inflammasomes are composed of three components: cytosolic pattern recognition receptors (PRRs, also known as inflammasome sensors), the adaptor ASC (apoptosis-associated spec-like protein containing a CARD), and the cysteine protease caspase-1. So far, five PRRs (NLRP1, NLRP3, NLRC4, Pyrin, and AIM2) have been identified as inflammasome-formation 18, 19. Given its potential role in several human diseases, the NLRP3 inflammasome has been the object of extensive research among these inflammasomes, which converts pro-caspase-1 into caspase-1. Activated caspase-1 cleaves the full-length GSDMD into GSDMD-N and GSDMD-C. GSDMD-N oligomerizes and boosts cell membrane pore formation by binding to membranes and destroying the stability of the membranes 15. Moreover, caspase-1 processes the pro-inflammatory cytokines, including pro-IL-1β and pro-IL-18 to be IL-1β and IL-18, accompanied with the release of other cell contents (such as lactate de-hydrogenase (LDH) and HMGB1) through the cell membrane pore, resulting in cell swelling and pyroptosis 6, 20.

In the non-canonical pyroptosis pathway, Shao et al. found that bacterial lipopolysaccharide (LPS) (or host-derived oxidized phospholipids) entered the cytoplasm through Toll-like receptors 4(TLR4) /MD2/CD14 signal pathway and bacterial outer membrane vesicles, and then directly activated caspase-4/5/11 21, 22. The activation of caspase-11 or caspase-4/5 does not require the PRR-mediated inflammasome. Activated caspase-4/5/11 can cleave GSDMD to GSDMD-N directly, but they are not accompanied by the cleavage of inflammatory factors pro-IL-1β and pro-IL-18 15, 23. However, they are able to mediate the maturation and secretion of IL-1β/ IL-18 through the NLRP3/caspase-1 pathway in some cells such as macrophages, leading to the inflammatory response 24. This also suggests that other members of caspase are involved in the inflammatory process by promoting caspase-1 activation 25. Therefore, the canonical and non-canonical pathways of pyroptosis are different in their induced signaling and initiation process, but eventually form pores on the plasma membrane (PM) by GSDMD and trigger pyroptosis through the inflammatory caspase.

In addition to the above two pathways, a caspase-3 dependent pathway has been revealed recently. In the case of administration of chemotherapeutic agents (e.g., DNA binding / modified compounds such as doxorubicin, cisplatin or topoisomerase inhibitors: etoposide, CPT-11, etc.), caspase-3 is activated to induce cleavage of GSDME, resulting in significant pyroptosis.

The morphology of cells changes obviously during pyroptosis. Swelling of cell membrane before rupture can be observed under scanning electron microscopy, and many bubble-like protrusions appear on the surface of cell membrane. Pyroptosis can be identified with dyes of lower molecular weights, such as 7-amino actinomycin (7-AAD), propidine iodide (PI), and ethidium bromide (EtBr) 26. In addition, there is a very specific type of DNA damage at the early stage of pyroptosis, which is positive for dUTP nick end labeling (TUNEL) staining. Recently, it has been found that in some cases, activated GSDMD does not always occur membrane rupture and pyroptosis. Macrophages, dendritic cells, and neutrophils are able to survive in the cleavage of GSDMD cleavage activated by inflammasomes, which are defined as cell hyperactivation 27, and it can be distinguished by detecting lactate dehydrogenase in cell culture supernatant. The release of cytokines can locally induce large-scale inflammatory response at the site of infection, causing cell death. Dead cells no longer participate in any immunomodulatory activities. If pyroptosis is balanced by the excessive activation of the cells described above, then the scorched cells will continue to secrete inflammatory factors and constantly affect immunomodulatory events. However, the detailed mechanism determining when GSDMD cleavage triggers scorching or hyperactivation is still unclear.

### Multiple Lipid Classes: Different Effects in Pyroptosis

Lipids such as cholesterol, together with Glycolipids and phospholipids, not only function as a major component of biological membranes, but also serve as the supply and storage of energy 28. Fatty acids (FAs) are the fundamental components of complex lipids such as phospholipids and glycolipids, which can be esterified with glycerol to form triglycerides and stored in lipid droplets. Under energy stress conditions, they can be oxidized and hydrolyzed by FA to produce ATP. In addition, lipids also play significant roles as signaling molecules. Cholesterol and oxysterol can activate downstream gene expression through sterol regulatory element-binding protein (SREBP). Phospholipases (PLC, PLD, and PLA) can catalyze the formation of bioactive second messenger molecules including diacylglycerol, lysophosphatidic acid, and arachidonic acid (AA) 29, 30. These different species of lipid molecules play key roles in regulating the fundamental processes of life, from energy storage to cell membranes formation to signal transduction Herein, we discuss these molecules and their derivatives that are produced in lipid metabolism and are associated with pyroptosis.

#### Cholesterol Derivatives and Pyroptosis

Intracellular cholesterol is maintained through de novo synthesis and exogenous uptake. Exogenous cholesterol follows an intricate pathway and then converts into free cholesterol in cells. However, excess free cholesterol may be toxic, which can cause pyroptosis both *in vivo* and *in vitro*. Specifically, overloading free cholesterol induces the expression of sphingomyelin synthase 1 (SMS1) in hepatocytes, and the diacylglycerol (DAG) produced by SMS1 activates protein kinase Cδ (PKCδ) and NLR family CARD domain-containing protein 4 (NLRC4) inflammasome to induce pyroptosis, thus triggering the development of non-alcoholic steatohepatitis (NASH) 30. Elevated free cholesterol can also activate endoplasmic reticulum (ER) stress by stimulating mSREBP1 in intervertebral disc degeneration, thereby inducing pyroptosis in nucleus pulposus cells 31.

Free cholesterol is transported from late lysosomes to other subcellular organelles, such as PM and ER 32. When cellular cholesterol trafficking is blocked, the cholesterol in PM decreases, resulting in the inhibition of NLRP3 assembly and the reduction of caspase-1, IL-1β and IL-18, eventually suppressing pyroptosis 33. However, another study has shown that when the cholesterol content in PM reached 30%, the binding, oligomeric assembly, and pore formation of GSDMD-N could be reduced 34. Generally, pyroptosis requires the N-terminal of the GSDMs to form pores in PM. These studies suggest that the content of PM cholesterol may directly affect pyroptosis through inflammasomes or combination with GSDMD. The relationship between PM cholesterol and pyroptosis is worth further exploration in the future. In the ER, cholesterol can be esterified. But the supersaturation of unesterified cholesterol in cells may cause precipitation of cholesterol crystals (CHCs) in the vascular wall, which is coincide with the earliest recruitment of inflammatory cells 35, 36. Hypercholesterolemia promotes the formation of CHCs and causes coronary endothelial cell damage *in vivo* and *in vitro* through NLRP3 activation. However, pretreatment with caspase-1 or HMGB1 inhibitors could significantly reverse endothelial-dependent vasodilation injury 37. A study has shown that the optimum dose of CHCs to induce pyroptosis was 0.5 mg/ml in human umbilical vein endothelial cells (HUVECs) 38. One of the critical mechanisms of CHCs-stimulated pyroptosis is lysosomal rupture. CHCs cause lysosomal damage, allowing lysosomal contents such as cathepsin B (CTSB) to penetrate into the cytoplasm and activate the NLRP3 inflammasome 39. On the other hand, CHCs in areas of plaque necrosis can stimulate the overproduction of mitochondrial reactive oxygen species (mtROS), thereby inducing the activation of NLRP3 inflammasome and caspase-1 2, 36, 38. ROS have been considered to play an important role in NLRP3 inflammasome activation and pyroptosis 40. Colchicine can suppress the uptake of CHCs by endothelial cells, and attenuate the occurrence of NLRP3 inflammasome and pyroptosis by inhibiting mtROS production and oxidative stress through the AMP-dependent kinase (AMPK) / Sirtuin1 pathway 38. The detailed mechanism of CHCs-induced pyroptosis remains unclear. In sum, these findings suggest a critical role for CHCs in the activation of pyroptosis.

Cholesterol can also be converted to oxysterols by chemical oxidation, such as 24(S)-hydroxycholesterol (24(S)-OHC), 27-OHC, 7β-OHC, and 25-OHC 41. Oxysterols can cause many types of cell death, such as apoptosis, autophagy, and necrosis 42. Recent studies have shown that oxysterol can also induce pyroptosis 43. 25-OHC promotes P2X7 receptor-dependent pyroptosis in skin cells, leading to the emergence of skin degenerative diseases 43. P2X7 receptor (P2X7) is a ligand-gated ion channel activated by extracellular ATP, which can assemble NLRP3 inflammasome 44. Another study also showed that ATP released by Pannexin-1, a non-selective and large-pore channels, activated P2X7 to allow small cations including K^+^ and Na^+^ to cross the PM and mediate pyroptosis 45. However, It has been found that in murine macrophages, 25-OHC can reduce the cellular content of sterols, IL-1β transcription and extensive inhibition of the inflammasome activation by antagonizing SREBP processing, thereby achieving an anti-inflammatory effect 46, which may trigger a potential inhibition of pyroptosis. In addition, excessive intracellular accumulation of 27-OHC induces lysosome dysfunction in co-cultured SH-SY5Y cells and C6 cells, and changes the levels of lysosome protein. CTSB is leaked through lysosomal membrane permeabilization (LMP) into the cytosol and induces NLRP3-dependent neuronal pyroptosis, causing neurodegenerative diseases such as Alzheimer's disease 47.

#### Fatty Acids and Pyroptosis

Fatty acids (FAs) are the hydrocarbon components of most lipids, usually with an even number of carbon atoms, ranging from 2-26. According to whether the hydrocarbon chain is saturated, fatty acids can be divided into saturated (SFA), monounsaturated (MUFA), and polyunsaturated fatty acids (PUFA). PUFAs are divided into two categories: omega-3 (ω-3) PUFAs and ω-6 PUFAs. FAs can also be divided into three types according to their amino acid chain lengths: short-, medium-, and long-chain FAs (SCFAs, MCFAs, and LCFAs, respectively 48.

Different FA types have distinct effects, even opposite influences. For obesity-related osteoarthritis, diet rich in SFA, MUFA, and n-6 PUFA can activate the TLR4/NF-κB signaling pathway in articular cartilage, which in turn upregulates the NLRP3 inflammasome, thereby inducing pyroptosis 49. Excessive free fatty acids (FFAs) induce tissue injury by activating pyroptosis, which is usually used to establish the cell model of NASH 50-53. Palmitic acid (PA) is the most dominant SFA in diet and is the main FFAs in plasma 54. Pyroptosis is the primary type of PA-induced cell death, which is related to activation of various protein kinases, ER stress, and recruitment of macrophages 55, 56. The combination of these factors leads to inflammasome-related cell death. PA induces NLRP3 mediated pyroptosis in HepG2 cells *via* ER stress 54. PA-induced lipotoxicity also causes mitochondrial damage and the release of mtDNA into the cytosol, thus triggering the cyclic GMP-AMP synthase (cGAS) - stimulator of interferon genes (STING) signaling pathway, which switches on the initiation of NLRP3 inflammasome-dependent pyroptosis in cardiomyocytes, promoting myocardial hypertrophy 57. In addition, higher levels of PA promote the polarization of macrophage M1, and upregulate the expression of cathepsin S (CTSS) through the transcription factor interferon regulatory factor 5 (IRF5). CTSS is transported in the exosomes, which can upregulate caspase-1 and trypsinogen in acinar cells, thus promoting pyroptosis and pancreatic tissue damage 58, 59. Besides, PA can enhance the endocytosis of LPS produced by gram-negative bacteria in the intestines of mice into intestinal neurons in a lipid raft-dependent manner, thus promoting LPS cleaving caspase-11 to regulate pyroptosis through non-classical pathway, eventually leading to the loss of enteric neuronal and enteric motility disorder 60. In sum, SFAs, especially PAs, can activate inflammasome-dependent pyroptosis in different ways.

In addition, many FAs can inhibit pyroptosis, thereby alleviating tissue damage. The diet rich in n-3 PUFA has anti-inflammatory and anti-pyroptosis effects. Docosahexaenoic acid (DHA), as a representative of ω-3 PUFA, is a potent inhibitor of both caspase-1 activation and IL-1β secretion 61, 62. DHA at a physiologically relevant concentration (less than 50 µM) is capable of attenuating pyroptosis 63, 64. DHA suppresses TLR4/NF-κB and NLRP3/caspase-1/GSDMD signaling pathways, thereby attenuating osteoarthritis 49. Further studies have shown that the anti-pyroptosis effect of DHA is to inhibit the assembly of NLRP3 inflammasome by promoting the interaction between G protein-coupled receptor (GPR) 120 and NLRP3. GPR120 is a PUFA receptor that mediates anti-inflammatory effects *via* PM internalization into the cytoplasm 29. The activation of GPRs causes the binding of β-arrestin2 to GPRs, and subsequent internalization of β-arrestin2, which then attaches to NLRP3 and prevents the assembly and activation of NLRP3 inflammasome 65-67. Meanwhile, DHA inhibits hypoxia/restoration (H/R)-induced injury by suppressing pyroptosis of hepatocytes induced by liver I/R injury *in vivo* and *in vitro* through the phosphatidylinositol-3-kinase /protein kinase B (PI3K/Akt) pathway 68. DHA can also exert an anti-inflammatory activity in the treatment of acute keratitis by alleviating the non-canonical pyroptosis 69. However, more than 50 μM of DHA can play a pro-inflammatory role. DHA (200 µM) at a higher concentration (within the physiological dose range) induces Bv-2 cell pyroptosis through 12-lipoxygenase (12-LOX). In detail, 12-LOX can produce one or more metabolism and activate a pro-inflammatory cell death program 63, 70. Similarly, all-trans retinoic acid (ATRA) secreted by hepatic stellate cells (HSC) can bind to retinoic acid receptors in KCs and activate the transcription activity of NLRP3. In addition, ATRA can also block autophagy, lead to excessive accumulation of ROS, and then activate NLRP3 inflammasome, thus inducing the pyroptosis of macrophages 71. In addition, a study has shown that DHA can cause pyroptosis of triple-negative breast cancer cells MDA-MB-231 72. Treatment with 200μM of DHA in breast cancer cells can lead to NF-κB translocation, caspase-1 and GSDMD activation, IL-1β secretion, HMGB1 translocation, pore membrane formation, and loss of membrane 72. It is noteworthy that DHA has no significant effect on human non-cancerous mammary epithelial cells MCF-10A or PBMCs, indicating that this fatty acid has cytotoxicity only on cancer cells 72. Therefore, ω-3 supplementation during therapy of breast cancer patients can be used as a new therapeutic strategy.

In addition to DHA, the metabolite short chain fatty acids (SCFAs) of gut microbiota including propionate (C3) and butyrate (C4) have been reported to be beneficial for pyroptosis and treatment of wear particle-induced osteolysis, in which C4 instead of C3 requires the GRP109a receptor in this process 73. 10-hydroxy-2-decenoic acid (10-HAD), the major fatty acid in royal jelly, can inhibit pyroptosis and treat ulcerative colitis 74. NLRP3 mediated pyroptosis induced by PA can be antagonized by oleic acid (OA), one of MUFAs 54. Chondrocytes treated with MUFAs showed the down-regulation of TLR4/NF-κB and NLRP3/caspase-1/GSDMD signaling pathways, while a MUFA-enriched high-fat diet stimulates the expression of TLR4/NF-κB and NLRP3 inflammasome proteins. The contradictory results of MUFAs *in vitro* and *in vivo* may be due to the fact that the high-fat diet rich in MUFA contains not only MUFAs, but also other pro-inflammatory FAs, such as SFA and n-6 PUFA. These FAs can weaken or even reverse the effects of MUFAs, resulting in the contradiction between the experimental results *in vitro* and *in vivo* 49.

#### Phospholipids and its Oxidative Derivatives and Pyroptosis

Phospholipids (PLs) are amphiphilic lipids that exist in the cell membranes of all plants and animals. They are arranged in lipid bilayers and include two major categories, glycerophospholipids, and sphingolipids. The PLs in most cell membranes are glycerophospholipids, which are composed of FA esterified into the glycerol back bone, phosphate groups, and hydrophilic residues (such as choline). Glycerophospholipids can be further divided into phosphatidylinositol (PI), cardiolipin (CL), phosphatidylserine (PS), phosphatidylcholine (PC), and phosphatidylethanolamine (PE) according to the substituents. The GSDMD-N possess lipid-binding and regulatory activities. GSDMD-N has the strongest binding with CL (a kind of mitochondrial and bacterial lipid) and phosphatidylinositol phosphates (PIPs), the phosphorylated products of PI 75, 76. This powerful combination enables GSDMD-N to locate the inner leaflet of the PM, form membrane-disrupting pores, and execute pyroptosis. And the same combination also exists on regulatorof cell death-1(RCD-1), a remote homolog of the N-terminal domain of gasdermin 77. phosphatidylinositol (4,5) bisphosphate (PIP2) and its synthetic precursor phosphatidylinositol-4-phosphate (PI4P) are the two main PIPs in PM. Decreasing their concentration can reduce the binding and oligomerization of GSDMD-N 16, 78. The accumulation of PI4P can potentiate the activation of NLRP3 inflammasome. In detail, NLRP3 is recruited into the dispersed trans-Golgi network (dTGN) through ionic bonding between its conserved polybasic region and negatively charged PI4P, and then interacts with ASC to activate the downstream signal cascade 79, 80. It has been shown that the dTGN is of endosomal origin. The endosomal accumulation of PI4P further impairs the trafficking of endosome to TGN 81.

Although the combination of GSDMD-N and PS is weak 75, GSDMD-N can also form oligomeric pores by binding with PS 82. Another study has shown that oligomeric pores mediated calcium influx, which induced PS transfer from inner leaflets to outer leaflets through transmembrane protein 16F, a calcium-dependent phospholipid scramblase 83. The asymmetric distribution of glycerophospholipids across the inner and outer leaflets of the PM is crucial for cellular integrity and signal transduction 84. Actually, GSDMD-N does not bind directly with PE or PC (the major lipids on both plasma membrane leaflets) 75. However, polyene PC, a clinical practice commonly used in the treatment of fatty liver, can reduce the expressions of GSDMD-N and other pyroptosis-related proteins 85. PC can be decomposed to lysophosphatidylcholine (LPC) under the catalysis of phospholipase A2 (PLA2). It is reported that LPC, can induce pyroptosis in human monocytes and human endothelial cells. LPC can also induce foam cell formation through LD biogenesis, which depends on the activation of NLRP3/caspase-1 86. Taken together, alteration of the phospholipid composition or distribution in the membrane directly affects the combination of GSDMD-N and lipids, which may be a potential strategy for inhibiting pyroptosis.

1-palmitoyl-2-arachidonoyl-snglycero-3-phosphorylcholine (PAPC) is a class of natural phospholipids. Under the condition of inflammation or oxidative stress, the side chains of polyunsaturated fatty acids in phospholipids are oxidized and modified to produce oxidized PAPC (oxPAPC). OxPAPC is usually present in dead cells, with concentrations of 10-100 μM at the site of injury 87. Similar to CHCs, OxPAPC is found in the early stage of atherosclerosis in mice and accumulated in the blood vessels of foam cells 36, 86, indicating that they contribute to inflammation in the early stage. However, unlike CHCs, oxPAPC exhibits anti-inflammatory effects and inhibits pyroptosis during gram-negative bacterial sepsis 88. This is due to the competitive combination of oxPAPC and LPS. LPS activates TLR4, triggers transcription on the cell surface, and activates caspase-4/11 in the cytosolic, resulting in pyroptosis 60. OxPAPC not only antagonizes TLR4, but also directly binds to caspase-4 and caspase-11, and competes with LPS, thereby inhibiting LPS-induced pyroptosis 88. Interestingly, compared with LPS, the combination of the above oxPAPC will not promote pyroptosis due to the unique positively charged amino acid residues 87. Moreover, oxPAPC induces an enhanced activation state of dendritic cells, called “hyperactive”, and then induces potent adaptive immune responses 87. Therefore, oxPAPC and its derivatives may provide a basis for therapies that target pyroptosis in gram-negative bacterial sepsis.

#### Low-Density Lipoprotein and its Oxidative Derivatives and Pyroptosis

Low-density lipoprotein (LDL) is a complex particle containing protein and lipids, and its outermost layer is surrounded by a lipid core and monomeric protein ApoB-100. LDL can induce inflammasome activation 89, but there are no reports related to pyroptosis LDL contains polyunsaturated fatty acids. When the antioxidant activity of LDL is impaired, ROS and reactive nitrogen species result in lipid oxidation to produce oxidized LDL (ox-LDL) 90. Ox-LDL is the main factor to promote foam cell formation and atherosclerosis, which may be one of the reasons why ox-LDL is used as an inducer in almost all studies on pyroptosis in atherosclerosis 91.

Ox-LDL can trigger pyroptosis directly or indirectly. On the one hand, ox-LDL can be directly recognized by TLR4, which can activate a series of downstream signals, such as NF-κB p65 phosphorylation, to promote transcription of pro-IL-1β and pro-caspase-1 92, 93. In addition, ox-LDL activated pyroptosis in primary human aortic EC through non-canonical NF-κB pathway that upregulated the transcription factor IRF-1 through RelB/p52. IRF-1 interacted with the GSDMD promoter at -526/-515 and the caspase-1 promoter at -11/10 to promote the expression and caspase-1-mediated activation of GSDMD 94. In addition, ox-LDL can also assist NLRP3 inflammasome assembly in various indirect ways. Ox-LDL can induce mitochondrial dysfunction and ROS release through multiple pathways, resulting in subsequent pyroptosis. Specifically, ox-LDL can down-regulate tet methylcytosine dioxygenase 2 (TET2) 95, thus reducing mitochondrial dysfunction 96. Zhaolin et al. showed that ox-LDL might induce EC pyroptosis and promote the development of atherosclerosis by regulating miR-125a-5p/ TET2 pathway 95. TET2 can inhibit the methylation of ubiquinol-cytochrome c reductase core protein 1 (UQCRC1), a subunit of mitochondrial complex III. Deletion of UQCRC1 can lead to excessive ROS production 97. Similarly, fibroblast growth factor 21 also inhibits ox-LDL-induced pyroptosis through the TET2-UQCRC1-ROS pathway 98. The proprotein convertase subtilisin/kexin type 9 (PCSK9) is an important protein involved in lipid metabolism and AS. Ox-LDL induces the pyroptosis of HUVECs in a concentration-dependent manner through PCSK9/UQCRC1/ROS pathway. PCSK9 can downregulate UQCRC1 expression and mediate ox-LDL-induced pyroptosis of HUVECs 99. Furthermore, UQCRC1 can be directly down-regulated by ox-LDL, and the silence of UQCRC1 aggravates HUVEC pyroptosis and the damage of mitochondrial function. TET2 is also an inhibitor of succinate dehydrogenase B (SDHB) and its deletion leads to the up-regulation of SDHB expression and activity by reducing the recruitment of histone deacetylase 2 100. Overexpression of SDHB in HUVECs impairs mitochondrial function, increases ROS level, and enhances pyroptosis, while knockout of SDHB can resist HUVEC pyroptosis 101. These studies indicate that ox-LDL can induce oxidative stress and mitochondrial disorder through a variety of ways, thus leading to the production of ROS 95. Undoubtedly, the elevated ROS also disrupts mitochondrial functions 99. Through this interaction and regulation, the participation of ROS is considered as the driving force of pyroptosis 102. ROS not only serves as an efficient trigger of the NLRP3 inflammasome, but also directly promotes GSDMD cleavage in pyroptosis, and up-regulates the oxidative modification of cysteine in GSDMD 102. Therefore, ROS is a triggering factor that activates NLRP3 inflammasomes agent and "effector" molecule 103.

Ca^2+^ influx is the upstream regulator of NLRP3 inflammasome activation. Studies have shown that ox-LDL induces the closure of macrophage K^+^ channels to open Ca^2+^ channels 104, and upregulates the expression of calcium-sensing receptor (CaSR) in rat aortic VSMCs in a time and dose-dependence manners to promote Ca^2+^ influx 105. Sebastian Rühl et al. found that the influx of Ca^2+^ through GSDMD pores was served as a signal for cells to initiate membrane repair by recruiting the endosomal sorting complexes required by transport (ESCRT) machinery to damaged membrane areas, such as the PM. Inhibition of the ESCRT-III machinery strongly exacerbates the activation of canonical or noncanonical inflammasome-dependent pyroptosis as well as the release of IL-1β in both human and murine cells 106. These studies suggest that ox-LDL may induce pyroptosis by promoting Ca^2+^ influx, and the rupture of membrane pores after pyroptosis is due to the Ca^2+^ influx activating ESCRT to repair cell membranes, and prevent the amplification of inflammation followed pyroptosis.

Ox-LDL can also induce K^+^ efflux via the activation of big conductance Ca^2+^-activated K^+^ channels (BKCa). In addition, P2X7 generates channels to promote K^+^ efflux 107. Definitely, K^+^ efflux is an essential upstream factor of caspase-1 activation. High levels of extracellular K^+^ can block the inflammatory responses induced by ox-LDL in HUVECs 108, while low concentrations of intracellular K^+^ are sufficient to trigger NLRP3 inflammasome 18. Ox-LDL up-regulates mixed lineage kinase domain-like (MLKL) protein, which activates NLRP3-induced pyroptosis by stimulating intracellular K^+^ efflux. MLKL-induced caspase-1 activation and IL-1β maturation can be abolished by NLRP3 specific inhibitor MCC950 108.

Pyroptosis is a form of pro-inflammatory cell death. Conversely, autophagy is a cell survival mechanism, which allows cells to survive by adapting to stress. There two cell processes are important components of immune regulation. In particular, the activity of autophagosome plays a pivot role in regulating cell deaths, and blocking autophagy promotes the pyroptosis of ox-LDL-treated macrophages *via* the p62/Nrf2/ARE axis 109. Mitochondrial receptor NIX inhibits ox-LDL-induced pyroptosis of human macrophage through autophagy and inhibition of caspase-1 activation 110. Ox-LDL-treated ECs exhibit increased pyroptosis mediated by myeloid cells trigger receptors1 (TREM-1) and decreased autophagy induced by Sirt6 111.

#### Synthetic Lipids and their Potential Application and Pyroptosis

Lipid nanoparticles (LNP) are multicomponent lipid systems that typically contain phospholipids, cationic lipids, cholesterol, and polyethylene glycolated lipids. Cationic lipids are key components of LNP, which can be permanently charged or acquire their charge at an acid pH and are also known as ionizable lipids. Cationic lipids possess a significant ability to stimulate the innate immune system, and are generally considered a safe alternative to viral vectors 112. The traditional type of lipid nanoparticles refers to liposomes. Several studies have demonstrated that the structure of lipids can affect these immune responses. Lipids with lysine head groups, ditetradecyl hydrophobic chains, and propyl or pentyl spacers, respectively, such as L3C14 and L5C14 liposomes, are most effective in activating the NLRP3 inflammasome 113. A previous study has shown that the arginine-based liposomes, such as A3C14 liposomes, can induce the most effective lysosomal disruption and NLRP3 inflammasome activation 114. Varying the concentrations of different lipid components in lipid nanoparticle formulations, the most notable of which are ionizable, cationic lipids, and cholesterol, can change their impact on the activation of NLRP3 inflammasomes, mainly due to the delay of endosomal rupture/fusing 115. In sum, the structural effect of cationic liposomes on the activation of NLRP3 inflammasome has provided insights into the application of lipid nanoparticles in improving immune response. The ability of cationic lipid nanoparticles can be exploited in gene therapy, anticancer or antiviral immunotherapies.

### Lipid Metabolism: A New Indicator of Pyroptosis

#### Key Enzymes in Lipid Synthesis: A Potential Aspects of Regulate Pyroptosis

Nearly 30 enzymatic processes convert acetyl CoA into cholesterol, such as mevalonate pathway 116. Inhibition of the mevalonate pathway leads to pyroptosis in Raw 264.7 monocyte cells 117. 3-hydroxy-3-methyl-glutaryl-coenzyme A reductase (HMGCR), as the key rate-limiting enzyme of the mevalonate pathway in cholesterol synthesis 118, is positively correlated with inflammation 119, 120 and interacts with NLRP3 121. HMGCR knockdown can reduce pyroptosis 121. Notably, the use of statins (a class of HMGCR inhibitors) including simvastatin and atorvastatin can attenuate pyroptosis 31. Mevalonate kinase (MK) is another important kinase in the mevalonate pathway, and its deficiency is associated with an auto-immune disease known as Mevalonate Kinase Deficiency (MKD). It has been shown that pyroptosis in MKD is a fundamental step to induce the inflammatory phenotype of MKD patients 122.

Fatty acid synthase (FASN) is the key enzyme to govern the de novo synthesis of fatty acids, which can convert acetyl-CoA, malonyl-CoA, and NADPH into SFA 123, 124. One study showed that the increase of FASN-mediated lipid synthesis of macrophages could enhance the caspase-1 activation mediated by NLRP3 and IL-1β expression through Akt and P38 MAPK pathways 125. Uncoupling protein-2 (UCP2) is a mitochondrial transport protein family located in the inner membrane of mitochondrial. It acts as a critical regulator of glucose-dependent de novo lipid synthesis *in vivo* and *in vitro*, and can up-regulate the expression of FASN in response to LPS and other stimuli 126. Another study revealed that knockdown or overexpression of UCP2 in hepatocytes could suppress or up-regulate FFAs-mediated pyroptosis respectively, which is manifested by the expressions of pyroptotic gene and accelerated cell death 127. This may be due to the activation of FASN mediates the increase of lipid synthesis, and ultimately promotes pyroptosis. SMS1 is an enzyme that generates sphingomyelin (SM) and DAG from de novo-synthesized ceramide 128. A recent study has shown that the overexpression of SMS1 in hepatocytes induces hepatocyte pyroptosis through the DAG-PKCδ-NLRC4 axis 30.

Conversely, pyroptosis also seems to affect lipid metabolism. GSDMD plays a key role in the pathogenesis of steatohepatitis by regulating lipogenesis. It has been found that knockout of GSDMD can reduce the expression of the lipogenic gene (such as FAS, PPARγ, SCD-1, and SREBP-1), while increasing the expression of lipolytic genes such as PPARα, CPT-1, ACO, LCAD, Cyp4a10 and Cyp4a14, to alleviate steatosis 129-131. In addition, caspase-4 can promote the synthesis and accumulation of fatty acids by up-regulating the expression of acetyl coenzyme A carboxylase, FASN, SREBP-1 and SREBP-2, and increasing the number of lipid droplets, and ultimately accelerate the progress of pancreatic cancer 132.

#### Lipid uptake is involved in Pyroptosis

Cholesterol can be transported through LDL binding to LDL receptor (LDLR) and internalization 133. A recent study has demonstrated that the expression of LDLR is down-regulated following acute cerebral ischemia, which exacerbated neuronal pyroptosis and inflammatory response by provoking the activation and recruitment of NLRP3 inflammasome, leading to the enlargement of cerebral infarct volume and the aggravation of neurological function defect 134.

The known FA protein transporters in PM include cluster of differentiation 36 (CD36) and FA-binding proteins (FABPs). CD36 is a scavenger receptor that can induce macrophage pyroptosis by regulating ox-LDL uptake 135, 136. In addition, the activation of inflammasome by *Porphyromonas gingivalis* (Pg) LPS in the oral cavity is mediated by CD36/scavenger receptor-B2 (SR-B2) and TLR2, and leads to systemic release of pro-atherosclerotic IL-1β, as well as induces macrophage pyroptosis. However, pyroptosis is reduced in the absence of CD36/SR-B2 137.

#### Lipid Storage: Hypertrophic Adipocytes induce Obese Adipocyte Pyroptosis

In a state of caloric excess, white adipose tissue mainly stores the surplus energy in the form of triglycerides. The volume of adipose tissue can be increased, in one of two main ways: hypertrophy or hyperplasia 138. The activation of NLRP3-dependent caspase-1 in hypertrophic adipocytes induces the pyroptosis of obese adipocytes, causing macrophage recruitment with metabolic consequences. These may be directly related to the large number of CHCs observed in transmission electron microscopy 139, 140. It has been reported that Nrf1 plays a key role in energy homeostasis by regulating lipid metabolism. Adipocyte-specific knockout of Nrf1 [Nrf1(f)-KO] in mice disturbs the expressions of lipolytic genes in adipocytes, resulting in white adipocyte hypertrophy, followed by severe adipose inflammation and pyroptosis 141, 142. Furthermore, overexpression of Nrf1 has been shown to inhibit tubular epithelial cell pyroptosis 143. Knockout of BMPR2 in adipocytes disrupts the phosphorylation of the lipid-droplet-coating protein (perilipin), and downregulates the lipolysis of white adipocytes, leading to subsequent caspase-1- dependent pyroptosis and inflammation 144. In addition to energy storage, adipose tissue is an active endocrine organ that regulates lipid metabolism by secreting adipokines like adiponectin (APN) 145. The secretion of APN in hypertrophic adipocytes is decreased 146, 147. APN can suppress lipopolysaccharide-induced pyroptosis by inhibiting forkhead transcription factor O 4 (FoxO4) in human aortic epithelial cells 148. In addition, leptin is also a hormone primarily derived from adipose tissue, and its plasma levels are correlated with fat storage 149. Leptin can modulate lipid metabolism in hepatocytes, resulting in hepatic steatosis 150. A study has shown that leptin exerts direct hepatocyte pyroptosis *via* ROS production/ER stress/autophagy induction/cathepsin B maturation/NLRP3 inflammasomes axis, leading to potential liver injury 151. Moreover, leptin can also trigger hepatocyte pyroptosis through CD8^+^ T lymphocytes. This effect relies on the Granzyme B released by CD8^+^ T lymphocytes 152. Altogether, these results suggest that failing lipid metabolism renders adipocytes vulnerable to pyroptosis, and prevention of adipocyte hypertrophy may improve the disease associated with pyroptosis.

#### The Interaction between Lipid Transport and Pyroptosis

Excess cholesterol in the ER drives the activation of the LXR transcription factors, which mediate cholesterol efflux by controlling the expression of the cholesterol export molecules ATP-binding cassette transporter 1 (ABCA1) and ATP-binding cassette subfamily G member 1 (ABCG1). ABCA1/G1-mediated cholesterol efflux is the initial step of reverse cholesterol transport (RCT). It is worth noting that ABCA1 also has certain anti-inflammatory effects. The lack of ABCA1/ABCG1 in cells leads to cholesterol accumulation and activation of NLRP3 inflammasomes 153, 154. A study has shown that ABCG1-knockout mice disturbed cholesterol metabolism and exacerbated pyroptosis after traumatic brain injury 155. ABCA1 expression can reduce the binding of GSDMD-N to PM, thus preventing cell lysis. Furthermore, ABCA1 is the floppase of PIP2 that transfers PIP2 from the inner to the outer leaflet of the PM, thereby reducing the content of PIP2 on the leaflets inside the PM 156. As the ligand of GSDMD-N fragments, PIP2 promotes cell rupture by transfection of GSDMD-N 75, 157, indicating that ABCA1 may indirectly inhibit GSDMD-induced membrane pore disruption and scorch death through PIP2. It is noteworthy that the pore formation caused by GSDMD can decrease the ability of cells to effectively transport cholesterol *via* the ABCA1-apoA1 pathway 157, which may increase cholesterol accumulation. Therefore, GSDMD-/- macrophages and mice can resist the reduction of RCT induced by pyroptosis.

Fatty acid binding protein 4 (FABP4) is a carrier protein of fatty acids, which is mainly expressed in macrophages and adipocytes and can regulate lipid metabolism. The increase of intracellular MUFA level in macrophages with FABP4 deletion leads to up-regulation of UCP2 expression, inhibition of ROS production, reduction of ER stress, and NF-κB activation and cytokine release attenuated, eventually leading to the anti-inflammatory phenotype in both animal and cell models 158, 159. The latest study further provides a mechanism, that is, the combination of FABP4 and MUFA can reduce the activation of silent mating type information regulation 2 homolog 1 (SIRT1) and the acetylation of p53. Pharmacological inhibition or genetic deletion of FABP4 in macrophages can deacetylate and inactivate p53 through SIRT1, which is the result of the loss of ASC expression. Lack of ASC prevents assembly of the NLRP3 inflammasome, GSDMD processing, and functional activation of pyroptosis 160. Together, these studies support a possible role for adipose tissue in promoting pyroptosis. Further studies are warranted to reveal molecular links between lipid droplet formation and pyroptosis inhibition.

#### Lipid Peroxidation: not the only Feature of Ferroptosis

Lipid peroxidation refers to the process of lipid peroxide generation mediated by free radicals on the cell membranes and organelle membranes 161. Glutathione peroxidase 4 (GPX4) is an antioxidant defense enzyme that can repair oxidative damage 162. Kang et al. showed that the inactivation of GPX4 gene increased lipid peroxidation-dependent caspase-11 activation, and aggravated GSDMD-mediated pyroptosis in macrophages as well as septic lethality in mice 163. And other studies have also demonstrated the ability of GPX4 to inhibit pyroptosis 164, 165, which may be related to lipid peroxidation. 5-Lipoxygenase (ALOX5) is an iron-containing and nonheme dioxygenase that catalyzes the peroxidation of PUFAs such as AA. Inhibition of lipid peroxidation by ALOX5 limits the activation of caspase-11 inflammasome and pyroptosis in macrophages, which provides a potential strategy for the treatment of sepsis 166. These results indicate that lipid peroxidation is closely related to pyroptosis. Interestingly, only lipid peroxides produced by the oxidation of phospholipids can induce GSDMD-N-mediated pyroptosis in BMDMs, instead of lipid peroxides formed by cholesterol and glycolipids in PM 163. Furthermore, lipid peroxidation of PM leads to phospholipase C (PLC) γ1 activation. PLCγ1, as an important second messenger, converts PIP2 to inositol 1,4,5-trisphosphate (IP3), resulting in the mobilization of ER calcium pool 167. On the one hand, PLCγ1-regulated calcium signal controls GSDMD-mediated pyroptosis by promoting GSDMD-N translocation to the PM 163, 168. On the other hand, the mobilization of intracellular calcium stores can also induce calcium flux and mtROS production, which triggers the activation of NLRP3 inflammasome and caspase-1, leading to IL-1β release as well as GSDMD-mediated pyroptosis 169.

#### Fatty Acid Oxidation

The main pathway of fatty acid catabolism is through fatty acid beta-oxidation (FAO), which is up-regulated under long-term fasting, exercise, or metabolic stress. Moon et al. have demonstrated that FAO promotes the activation of NLRP3 inflammasome through NADPH oxidase 4 (NOX4). Mechanistically, NOX4 acts as a source of cellular superoxide anion, which enhances the expression of carnitine palmitoyltransferase 1A (CPT1A) to promote the activation of NLRP3 inflammasome, and CPT1A is a key enzyme in the FAO pathway 170. Another study showed that NOX promoted the production of mtROS or self-derived ROS, and activated NLRP3 inflammasome. It was further found that NOX4 was positively correlated with the expressions of caspase-1 and GSDMD-N, and inhibition of NOX4 could prevent cardiomyocyte pyroptosis 171. Since NOX4-derived mtROS is important for regulating the expression of the CPT1A 170, it is believed that NOX4-derived mtROS may stimulate NLRP3 by activating CPT1A-modulated FAO, thus leading to pyroptosis.

## Conclusion

Pyroptosis is an inflammatory programmed death pathway. Numerous types of lipids, such as CHCs, ox-LDL, and SFAs, act as signal molecules to activate pyroptosis. Conversely, lipids such as UFAs can inhibit pyroptosis. In general, most of these lipids activate NLRP3-dependent pyroptosis by inducing mitochondrial dysfunction, ER stress, and lysosomal disruption, while other types of lipids directly activate GSDMD without relying on NLRP3. Pyroptosis is usually induced by LPS and ATP (or Nigericin) 172. But accumulating studies used lipids (such as ox-LDL and PA) to establish pyroptosis model, especially in the studies of cardiovascular diseases and NAFLD. Lipid metabolism is regulated by complex intracellular signal network and constitutes an essential part of cell fate regulation. Meanwhile, enzymes or proteins related to lipid metabolism also affect pyroptosis. For example, the GSDMD-N fragments cleaved by caspase-1 trigger pyroptotic macrophages in a PLC1-dependent manner.

Cholesterol in the endoplasmic reticulum is involved in the activation of NLRP3. Silencing NLRP3 and ASC in macrophages, CHCs are unable to release IL-1β demonstrated its ability to activate NLRP3 inflammasome. Cardiolipin is beneficial to the formation of NLRP3 inflammasome. SFAs, such as PA, stimulate TLRs and induce K^+^-dependent NLRP3 inflammasomes. By contrast, UFAs prevent SFAs-induced NLRP3 activation. In addition to these endogenous host-derived lipids, synthetic lipids (cationic and ionizable lipids) also have been described to regulate the activation of inflammasome. All of these have been discussed in detail in the review by Pizzuto M et al 173. It is easy to see the important role of lipids in the activation of NLRP3 inflammasome. To date, there are still some issues to be solved. The extent of pyroptosis caused by activation of NLRP3 inflammasome is still unclear, and how inflammasomes develop into pyroptosis requires further exploration. The relationship between synthetic lipids and NLRP3 may have potential roles in synthetic lipids and pyroptosis. Lipids can activate the occurrence of pyroptosis, but there are few studies on whether the large-scale inflammatory response caused by pyroptosis will affect lipid metabolism in turn, and thus the level of blood lipids. It is well known that the inflammatory response can aggravate the imbalance of lipid metabolism, promotes the accumulation and uptake of lipids, as well as inhibit efflux. Therefore, the alterations and status of lipid metabolism-related proteins, such as ABCA1/G1, ABCG5/8, PCSK9, etc. in the process of cytokinesis, deserve in-depth investigations. In addition, as a form of programmed death, pyroptosis plays a protective role, especially in immune defense, but the inflammatory factors released by excessive pyroptosis can lead to a certain degree of tissue damage.

It is exciting to note that inhibitors of the inflammasome-pyroptosis pathway have been identified. Several drugs targeting pyroptosis such as MCC950, VX-765, z-VAD-fmk, have been developed and validated *in vitro* cell culture studies and animal models of inflammation-related diseases *in vivo*, but prospective clinical trials are also required to potentially translate them into clinical practice. This is because the activation and action mechanism of pyroptosis is extremely intricate, and a consensus model has not been formed yet. Therefore, a large number of experiments, especially clinical trials, are still urgently required. Other potential drug candidates, such as autophagy inducers, antioxidants, and miRNA reagents, need further development. These studies will help to deepen the understanding of the pathogenesis of many diseases, and develop effective treatment strategies from the perspective of pyroptosis.

## Figures and Tables

**Figure 1:**
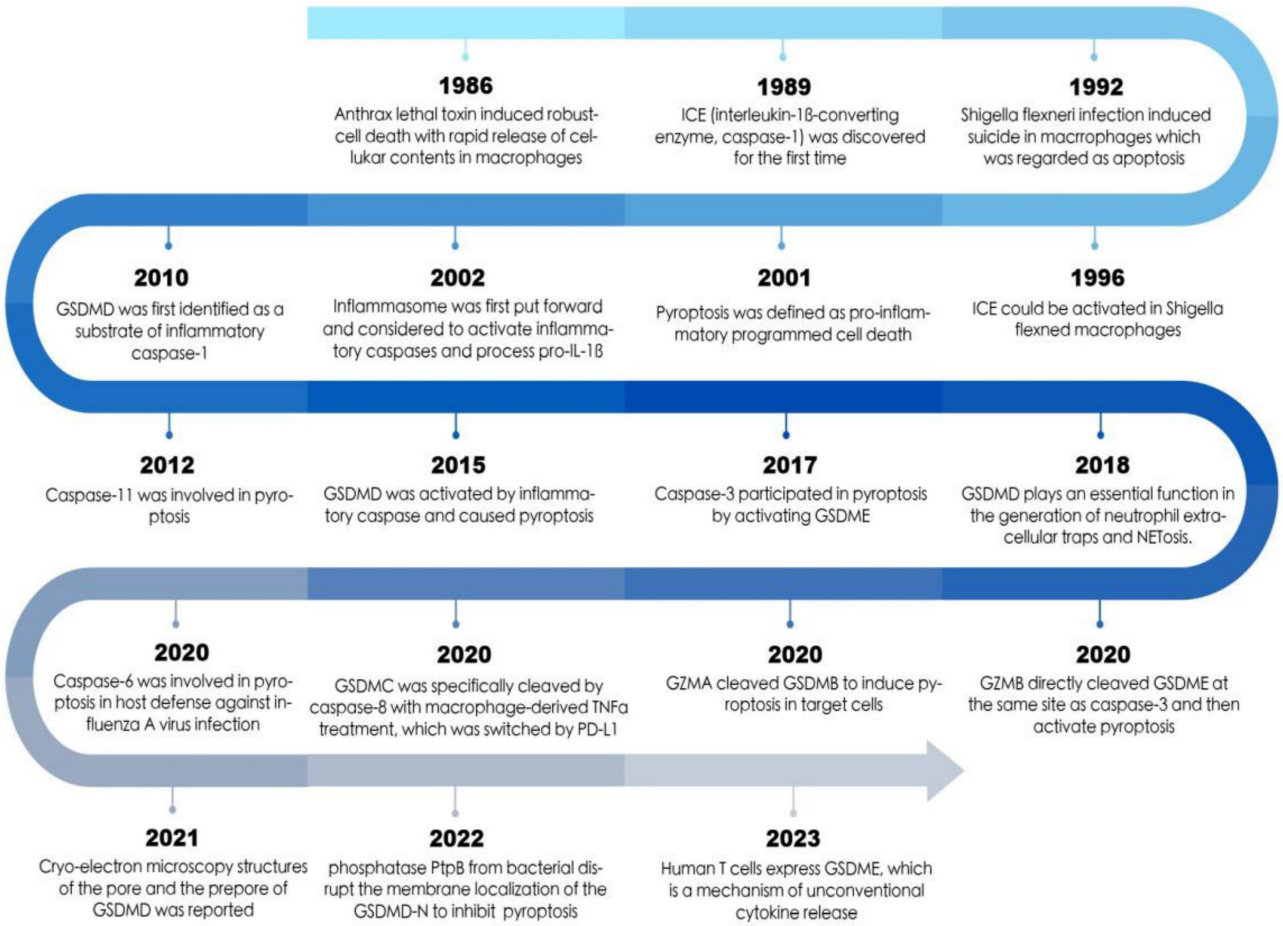
The timeline of pyroptosis

**Figure 2:**
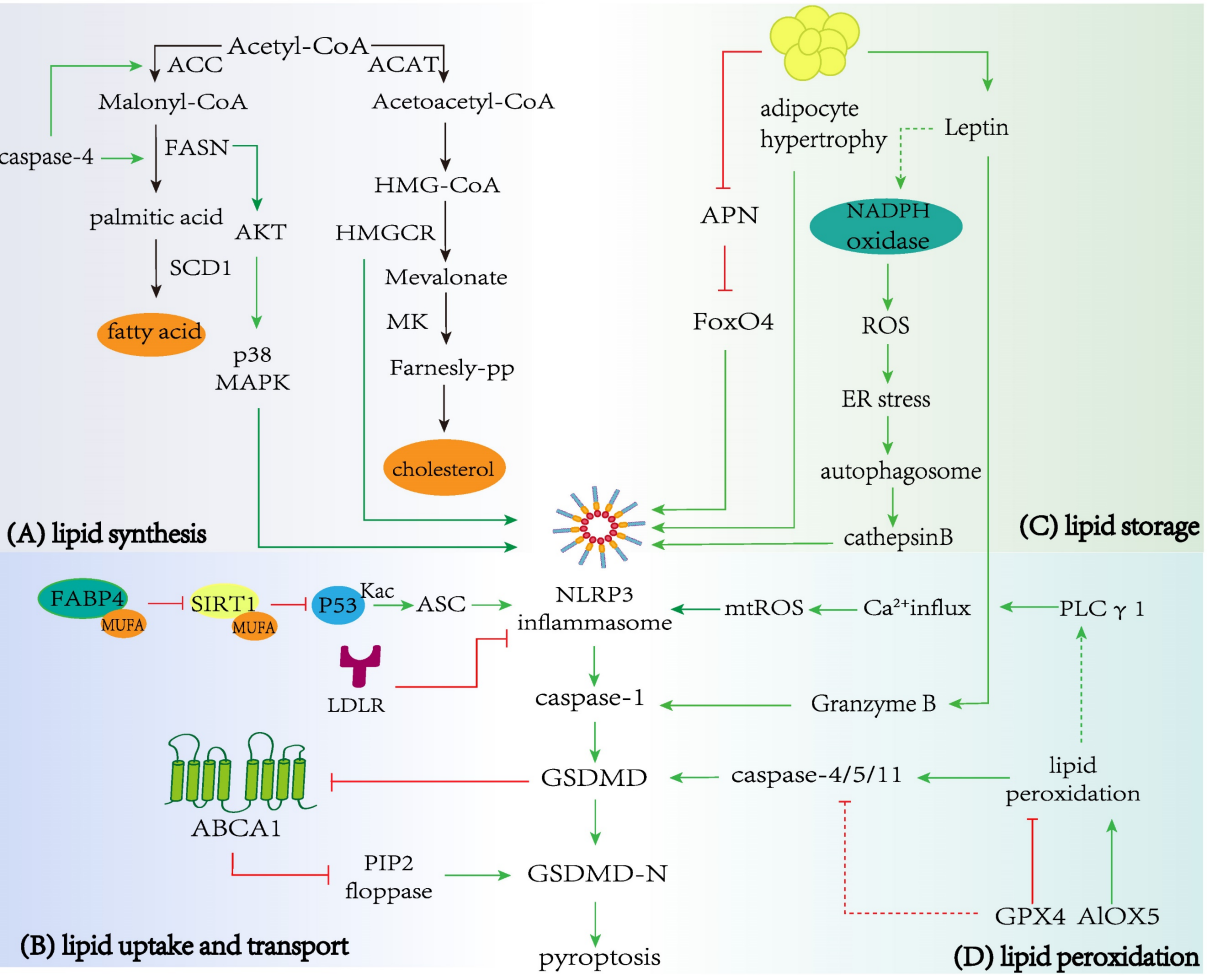
** Regulation of lipid metabolism in pyroptosis. A** Lipid synthesis and pyroptosis. Enzymes in cholesterol and fatty acid biosynthesis, including FASN and HMGCR can promote the occurrence and development of pyroptosis. In addition, the pyroptosis-related protein caspase-4 promote critical enzymes in fatty acid synthesis, including ACC and FASN. **B** Lipid uptake and transport with pyroptosis. LDLR and ABCA1 restrain NLRP3 inflammasome activation and prevent caspase-1 cleaving GSDMD to promote the release of the N-terminal domain, which executes pores formation and pyroptosis. The combination of FABP4 and MUFA can reduce activation of SIRT1 and acetylation of p53, promoting NLRP3-dependent pyroptosis. **C** Lipid storage and pyroptosis. Hypertrophic adipocytes can induce NLRP3-dependent caspase-1 activation and pyroptosis, and obese adipocytes also regulate the secretion of APN and leptin. APN and leptin promote pyroptosis *via* FoxO4 or ROS production/ER stress/autophagy induction/cathepsin B maturation axis respectively. **D** Lipid peroxidation and pyroptosis. GPX4 and ALOX5 inhibit or promote lipid peroxidation respectively, leading to caspase-11-dependent pyroptosis. Lipid peroxidation triggers NLRP3 inflammasome and caspase-1 activation by inducing PLC γ1 activation.

**Table 1:** *In vitro* and *in vivo* evidence of lipid and its metabolites regulate pyroptosis.

Category of lipid	*In vivo*	*In vitro*	Effect on pyroptosis	mechanism	Ref
CHCs	NLRP3^-/-^mice	Mouse primary coronary arterial endothelial cells	Promote	mtROS↑/NLRP3↑	40
Cholesterol	HFHCD-fed mice	hepatocyte	Promote	DAG↑/PKCδ↑/NLRC4↑	30
	HCD-fed rats	NP cells	Promote	mSREBP1↑/ER stress↑	31
27-OHC	NA	SH-SY5Y cells, C6 cells	Promote	LMP↑/CTSB↑/NLRP3↑/caspase-1↑	47
25-OHC	NA	NHEK,HaCaT cells,	Promote	P2X7R↑/NLRP3↑/caspase-1↑	43
PA	obesity-associated osteoarthritis mice	SW1353 chondrocytes	promote	TLR4↑/NF-κB↑ NLRP3↑/caspase-1↑/GSDMD↑	49
	NAFLD rat	HepG2 cells	Promote	ER stress↑/NLRP3↑	54
	hyperlipidemic pancreatitis mice	Rat pancreatic acinar cells	Promote	IRF5↑/CTSS↑/caspase1↑/GSDMD↑	59
	caspase-11-knockout mice, Western diet-fed mice	enteric neuronal cells	Promote	caspase-11↑/GSDMD↑	60
	DCM mice	Myocardial cells	Promote	cGAS↑/STING↑	57
DHA	Buffalo rat	Kupffer cells	Inhibit	PI3K↑/Akt↑/caspase-1↑	68
	Acute keratitis rats	Human corneal epithelial cells	Inhibit	Caspase-11↓/p30↓	69
	LPS-exposed mice	Kuppfer cells	Inhibit	GPR120↑/β-arrestin2↑/ NLRP3↓	29
	obesity-associated osteoarthritis mice	SW1353 chondrocytes	Inhibit	TLR4↓/NF-κB↓ NLRP3↓/caspase-1↓/GSDMD↓	49
	NA	murine microglia cells	Promote DHA (200µM)	12-LOX↑	63
	NA	MDA-MB-231 cells,4T1 cells	Promote DHA (200µM)	NF-κB translocation↑, caspase-1↑/GSDMD↑	72
OA	NAFLD rat	HepG2 cells	Inhibit	ER stress↓/NLRP3↓	54
Propionate、butyrate	C57BL/6J male mice	BMDMs、THP-1、Osteoclast differentiation	Inhibit	NLRP3↓/Caspase-1↓/GSDMD-N↓	73
10-HDA	ulcerative colitis mice	THP-1	Inhibit	NLRP3↓/Caspase-1↓/GSDMD-N↓	74
PI(4,5)P2	Gsdmd^-/-^ mice	HEK293T、Hela、iBMDM	Promote	GSDMD-N↑	75
PI4P	Gsdmd^-/-^ mice	HEK293T、HeLa	Promote	GSDMD-N↑	78
cardiolipin	NA	Hela cells、293T	Promote	GSDMD-N↑	16
phosphatidylserine	NA	Asymmetric plasma membrane composition	Promote	GSDMD-N↑	82
LPC	NA	THP-1、HUVEC	Promote	NLRP3↑/Caspase-1↑/GSDMD-N↑	86
oxPAPC	Casp1^-/-^, Casp11^-/-^, Tlr4^-/-^, Tlr3^-/-^, and Cd36^-/-^ mice	BM cells were flushed from femurs and tibias	Inhibit	TLR4↓/caspase4,11↑	88

## Abbreviations

AA: Arachidonic acid
ABCA1: ATP-binding cassette transporter 1
ABCG1: ATP-binding cassette subfamily G member 1
ALOX5: 5-Lipoxygenase
AMPK: AMP-dependent kinase
APN: adipokines like adiponectin
ASC: apoptosis-associated spec-like protein containing a CARD
ATP: Adenosine triphosphate
BMPR2: bone morphogenetic protein receptor type 2
CD36: cluster of differentiation 36
CHCs: cholesterol crystals
COPII: coat protein complex II
CPT1A: carnitine palmitoyltransferase 1A
CTSB: cathepsin B
CTSD: cathepsin D
DHA: Docosahexaenoic acid
ER: endoplasmic reticulum
EtBr: ethidium bromide
FABP4: Fatty acid binding protein 4
FABPs: FA-binding proteins
FAO: fatty acid beta-oxidation
FAs: Fatty acids
FASN: Fatty acid synthase
FoxO4: forkhead transcription factor O 4
GPR: G protein-coupled receptor
GPX4: Glutathione peroxidase 4
GSDMA: gasdemin A
GSDMB: gasdemin B
GSDMC: gasdemin C
GSDMD: gasdemin D
GSDME: gasdemin E
GSMD: Gasdermin
H/R: hypoxia/restoration
HFHCD: high-fat and high-cholesterol diet
HMGB1: high mobility group box 1
HMGCR: 3-hydroxy-3-methylglutaryl coenzyme A reductase
IL-1β: interleukin-1β
IP3: inositol 1,4,5-trisphosphate
LDH: lactate de-hydrogenase
LDL: Low-density lipoprotein
LPC: lyso-PC
LPS: lipopolysaccharide
LXR: Liver X receptors
MAPK: mitogen activated protein kinase
MK: Mevalonate Kinase
MKD: Mevalonate Kinase Deficiency
mtROS: mitochondrial reactive oxygen species
MUFA: monounsaturated
NASH: non-alcoholic steatohepatitis
NF-κB: nuclear factor kappa B
NOX4: NADPH oxidase 4
OA: oleic acid
ox-LDL: oxidized LDL
oxPAPC: oxidized PAPC
PA: palmitic acid
PAPC: 1-palmitoyl-2-arachidonoyl-snglycero-3-phosphorylcholine
PCD: programmed cell death
PCSK9: proprotein convertase subtilisin/kexin type 9
Perilipin: the lipid-droplet-coating protein
Pg: Porphyromonas gingivalis
PI: propidine iodide
PI3K/Akt: phosphatidylinositol-3-kinase/protein kinase B
PIP2: phosphatidylinositol(4,5)bisphosphate
PKCδ: protein kinaseCδ
PLC: phospholipase C
PLs: Phospholipids
PRRs: pattern recognition receptors
PUFA: polyunsaturated fatty acids
RCT: reverse cholesterol transport
ROS: reactive oxygen species
SDHB: succinate dehydrogenase B
SFA: saturated fatty acids
SIRT1: silent mating type information regulation 2 homolog 1
SR-B2: scavenger receptor-B2
SREBP: Sensor response element binding protein
TET2: Tet methylcytosine dioxygenase 2
TLR4: Toll-like receptor 4
TUNEL: dUTP nick end labeling
UCP2: Uncoupling proteins 2
UQCRC1: ubiquinol-cytochrome c reductase core protein 1
7-AAD: 7-amino actinomycin
12-LOX: 12-lipoxygenase
